# In response to nocturnal dipping profile in chronic kidney disease: Searching for underlying mechanisms in order to prevent adverse events

**DOI:** 10.14814/phy2.14674

**Published:** 2020-12-23

**Authors:** Jinhee Jeong, Jeanie Park

**Affiliations:** ^1^ Division of Renal Medicine Department of Medicine Emory University School of Medicine Atlanta GA USA; ^2^ Research Service Line Department of Veterans Affairs Health Care System Decatur GA USA

To the Editor:

We appreciate the interest in our study and the commentary by Dr. Theodorakopoulou and colleagues regarding our work (Jeong et al., 2020).

As noted in the commentary, our study was the first to demonstrate the link between nocturnal blood pressure (BP) profiles, elevated sympathetic nervous system (SNS) activation, and impaired vascular endothelial function in patients with chronic kidney disease (CKD; Jeong et al., 2020). Despite the high prevalence of non‐dipping BP patterns (Iimuro et al., 2013; Pogue et al., 2009) and its strong prognostic value for target organ damage and worse cardiovascular outcomes in patients with CKD (Choi et al., 2017; Jaques et al., 2018), there is a lack of knowledge in the pathophysiological mechanisms underlying non‐dipping in this population. Our study attempted to provide a groundwork for potential mechanisms to explain the abnormally elevated nighttime BP with multiple analytic approaches. Notably, we are the first to use direct, intraneural recordings of muscle sympathetic nerve activity (MSNA), the gold‐standard technique for measuring sympathetic nerve impulses in humans, to demonstrate higher SNS activity in CKD patients with non‐dipping BP patterns.

Our study population comprised of male and primarily overweight Black adults with CKD. As we noted in our manuscript, inclusion of only males is a limitation and future studies should be conducted in females with CKD. In terms of Black race, while we agree that our results may not be generalizable to White patients or other races, we feel that it was important to study Black adults with CKD, an understudied patient population that has a disproportionately higher rate of kidney disease in the United States (Centers for Disease Control & Prevention, 2019). Our patient population is representative of the general CKD population of the metro Atlanta area and the Southeastern US, and our studies contribute new mechanistic knowledge regarding physiologic underpinnings in these patients.

Collecting high‐quality MSNA recordings, 24‐h ambulatory BP data, and brachial artery flow‐mediated dilatation in 32 participants was no easy feat. The sample size is on the higher side for typical microneurographic studies, and we were able to detect significant group differences. Indeed, the majority of studies linking nocturnal non‐dipping with adverse cardiovascular outcomes in patients with CKD and hypertension have used the dichotomous grouping strategy (Choi et al., 2017; Jaques et al., 2018). We do agree that we were not powered to investigate multiple categories of diurnal BP profiles, and we think it would be very interesting to investigate the mechanisms underlying the morning BP surge as well as the role of salt sensitivity. It should be noted that in addition to group analyses, our primary analytic strategies included linear association, multivariable regression analysis, and mediation analysis among the primary outcomes. These additional analyses provide further evidence of a link between non‐dipping BP patterns, SNS activity, and endothelial function by providing numerical predictive estimates of MSNA and FMD for the entire range of nighttime BP and dipping ratios.

In summary, we appreciate the interest in our work and plan to expand our studies investigating autonomic and vascular mechanisms underlying aberrant diurnal BP patterns in CKD. One current limitation in the field is the inability to measure 24‐h SNS activity. While microneurography provides rigorous measurements of SNS activity in real time, its measurement is limited to the laboratory setting. Measures such as heart rate variability (HRV) can be obtained over 24 h; however, although HRV provides valid estimates of parasympathetic activity, HRV is not an accurate reflection of SNS activity. Studies designed to assess 24‐h patterns of SNS activity, perhaps through microneurographic recordings obtained during the daytime and at nighttime, or through the development of new techniques to estimate continuous SNS activity in an ambulatory setting, would add valuable insight into both normal and aberrant diurnal autonomic patterns and their relationship to nocturnal hypertension.

Sincerely,

Jinhee Jeong, Ph.D.

Jeanie Park, M.D., M.S.
